# Safety of Ramadan fasting in young patients with type 1 diabetes: A systematic review and meta‐analysis

**DOI:** 10.1111/jdi.13054

**Published:** 2019-04-26

**Authors:** Huai Heng Loh, Lee‐Ling Lim, Huai Seng Loh, Anne Yee

**Affiliations:** ^1^ Faculty of Medicine and Health Sciences University of Malaysia Sarawak Sarawak Malaysia; ^2^ Department of Medicine Faculty of Medicine University of Malaya Kuala Lumpur Malaysia; ^3^ Asia Diabetes Foundation Shatin, Hong Kong SAR China; ^4^ Clinical Academic Unit Newcastle University Medicine Malaysia Johor Malaysia; ^5^ Department of Psychological Medicine Faculty of Medicine University of Malaya Kuala Lumpur Malaysia

**Keywords:** Hyperglycemia, Hypoglycemia, Insulin pump

## Abstract

**Aims/Introduction:**

Although patients with type 1 diabetes are medically exempt, many insist on fasting during Ramadan. Multiple daily insulin injections (MDI), premixed insulin and continuous subcutaneous insulin infusion (CSII) are commonly used. To date, little is known about the safety of Ramadan fasting in these patients.

**Materials and Methods:**

We pooled data from 17 observational studies involving 1,699 patients treated with either CSII or non‐CSII (including premixed and MDI) regimen. The study outcomes were the frequencies of hypoglycemia, hyperglycemia and/or ketosis. Given the lack of patient‐level data, separate analyses for premixed and MDI regimen were not carried out.

**Results:**

The CSII‐treated group (*n* = 203) was older (22.9 ± 6.9 vs 17.8 ± 4.0* *years), and had longer diabetes duration (116.7 ± 66.5 vs 74.8 ± 59.2* *months) and lower glycated hemoglobin (7.8 ± 1.1% vs 9.1 ± 2.0%) at baseline than the non‐CSII‐treated group (*n* = 1,496). The non‐CSII‐treated group had less non‐severe hypoglycemia than the CSII‐treated group (22%, 95% CI 13–34 vs 35%, 95% CI 17–55). Of the non‐CSII‐treated group, 7.1% (95% CI 5.8–8.5) developed severe hypoglycemia, but none from the CSII‐treated group did. The non‐CSII‐treated group was more likely to develop hyperglycemia (12%, 95% CI 3–25 vs 8.8%, 95% CI 0–31) and ketosis (2.5%, 95% CI 1.0–4.6 vs 1.6%, 95% CI 0.1–4.7), and discontinue fasting (55%, 95% CI 34–76 vs 31%, 95% CI 9–60) than the CSII‐treated group.

**Conclusions:**

The CSII regimen had lower rates of severe hypoglycemia and hyperglycemia/ketosis, but a higher rate of non‐severe hyperglycemia than premixed/MDI regimens. These suggest that appropriate patient selection with regular, supervised fine‐tuning of the basal insulin rate with intensive glucose monitoring might mitigate the residual hypoglycemia risk during Ramadan.

## Introduction

Ramadan fasting is one of the five pillars of Islam and is obligatory for all healthy Muslim adults^1^. During this holy ninth lunar month, Muslims refrain from eating and drinking from dawn to dusk. However, if one is suffering from any medical condition that will be negatively affected by prolonged fasting, the patient is advised against fasting during Ramadan^2^.

In patients with diabetes mellitus, the fine adjustments of glucose homeostasis during fasting through glycogenolysis and gluconeogenesis with a concurrent rise in circulating glucagon and a fall in insulin level are lost, especially among those with type 1 diabetes mellitus^3^. These patients are also unable to mount an appropriate hormonal response to handle a state of prolonged nutrition deprivation^4^. Apart from patients with poorly controlled type 2 diabetes mellitus, the current guidelines recommend that patients with type 1 diabetes mellitus, even with well‐controlled disease, should not fast^5^. However, the landmark Epidemiology in Diabetes and Ramadan study showed that almost half of the patients with type 1 diabetes mellitus insisted on fasting during Ramadan^2^. In the recent Multi‐Country Retrospective Observational Study of the Management and Outcomes of Patients with Diabetes during Ramadan study involving 3,250 patients with type 2 diabetes mellitus (mean glycated hemoglobin [HbA_1c_] at baseline 7.6 ± 1.6%), >90% of patients fasted at least 15 days during Ramadan^6^.

The insulin regimens that are commonly used for patients with type 1 diabetes mellitus are multiple daily insulin injections (MDI), premixed insulin and continuous subcutaneous insulin infusion (CSII). Many healthcare providers face significant challenges of advising patients with type 1 diabetes mellitus who insist on fasting regarding the appropriate choice and adjustment of their insulin regimens, as well as the timing of breaking the fast during Ramadan^7^. Of note, many patients were found to fast without informing their healthcare providers or obtaining proper instructions and recommendations on appropriate management during Ramadan fasting^8^.

Against this background, the present meta‐analysis aimed to systematically review and analyze the safety of Ramadan fasting among patients with type 1 diabetes mellitus who were treated with either the CSII or non‐CSII (including MDI and premixed insulin) regimen. The primary outcome was the proportion of patients who developed either hypoglycemia, hyperglycemia or ketosis during Ramadan fasting. The secondary outcomes were: (i) the proportion of patients breaking the fast during Ramadan; and (ii) the changes in fructosamine and bodyweight before and after Ramadan.

## Methods

### Data sources and extraction

We carried out a systematic search of all English‐language medical literature published from inception up to January 2018 using PubMed, CINAHL, Ovid, Embase and Cochrane Library. We used the following Medical Subject Headings: “fasting”, “Ramadan”, “diabetes mellitus” and “type 1”. We also looked into the references of the selected articles. When the articles were not available or information of a study cohort was inadequate, we attempted to contact the respective authors by e‐mail to obtain the full articles and more detailed data. The titles and abstracts obtained through the electronic search were screened, followed by an analysis of the full text articles by two independent reviewers (HHL and HSL). All duplicates were removed. Wherever data were not provided numerically, they would be read off graphs. Data from eligible studies were extracted by HHL, and all extracted data were reviewed by LLL.

### Quality assessment

HHL and AY independently assessed the quality of the methodology and reporting of the studies using the Strengthening the Reporting of Observational Studies in Epidemiology (STROBE) statement. Any discrepancies were resolved by the third reviewer (HSL). The STROBE statement has a checklist of 22 items with information on the title, abstract, introduction, methods, results and discussion of the included articles. Among these, 18 items are common to cohort, case–control and cross‐sectional studies, whereas four items are specific to each of the three study designs. The STROBE statement facilitates the reviewers to critically appraise and interpret the included studies^9^.

### Ethical approval

This study complied with the Declaration of Helsinki. Given this study was a meta‐analysis, no prior ethical approval was required.

### Statistical analysis

#### Qualitative data

All abstracted information was tabulated. A qualitative meta‐analysis was carried out to summarize, compare and contrast the abstracted data.

#### Quantitative data

All analyses were carried out using StatsDirect version 2.7.9 (StatsDirect Ltd., Cambridge, UK). The presence of heterogeneity between studies was tested using the *I* ^2^ statistic, with an *I* ^2^ ≥ 70% indicating significant heterogeneity. If the *I* ^2^ was significant, we pooled the data with random‐effects models using the DerSimonian–Laird method. Conversely, we pooled the data with fixed effects models using either the Mantel–Haenszel or Rothman–Boice method. We also assessed the publication bias with the Begg–Mazumdar and Egger test. For dichotomous data, including the proportion of patients with hypoglycemia, hyperglycemia, ketosis and the need for breaking the fast, we estimated the odds ratio (OR) with 95% confidence intervals (CI) using the fixed effects models.

## Results

**Figure 1:**
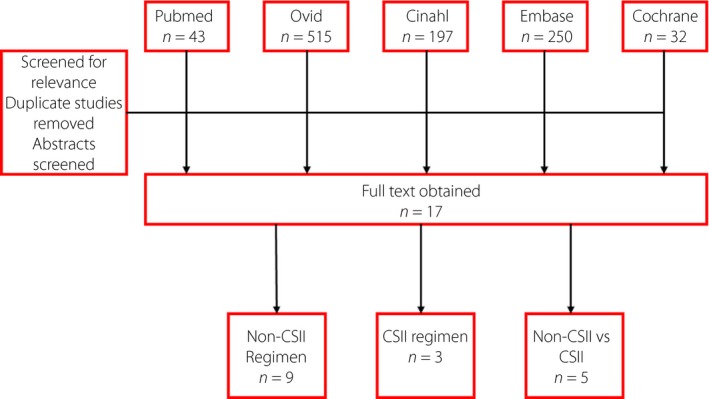
Study flow diagram. CSII, continuous subcutaneous insulin infusion.

Figure 1 shows the study selection process. Our initial search identified a total of 755 articles with 43 articles from PubMed, 197 articles from CINAHL, 515 articles from Ovid, 250 articles from Embase and 32 articles from Cochrane Library. After the screening of titles, abstracts and full texts, and elimination of duplicate publications, 17 articles were eligible for inclusion into the present meta‐analysis.

### Data synthesis

A total of 1,699 patients with type 1 diabetes mellitus were included, with the sample size ranging between five and 1,070. Nine studies involved patients who were treated with the non‐CSII regimen^2^, ^8^, ^10^, ^11^, ^12^, ^13^, ^14^, ^15^, ^16^, three studies involved patients treated with CSII^17^, ^18^, ^19^ and five studies compared patients treated with either a non‐CSII or CSII regimen^20^, ^21^, ^22^, ^23^, ^24^.

The characteristics of the included studies and baseline demographic data of these patients are summarized in Table 1. All studies evaluated the occurrence of hypoglycemia among patients who fasted during Ramadan. There were 10 studies that defined hypoglycemia using a cut‐off of either <4.4^24^, <4.0^13^, <3.9^14^, ^18^, ^19^, ^21^, ^23^, <3.5, <3.3^20^, <3.0^16^ or <2.9 mmol/L^8^. Another three studies defined hypoglycemia as either a need for breaking the fast^11^, ^17^ or the presence of symptoms of hypoglycemia, irrespective of the glucose level^12^. Among these 13 studies, seven of them also defined severe hypoglycemia as either requiring third‐party assistance, parenteral glucose/glucagon administration or hospitalization^8^, ^11^, ^14^, ^17^, ^19^, ^20^, ^24^.

**Table 1:** Characteristics of studies evaluating the safety of Ramadan fasting among patients with type 1 diabetes

Study	Site	Study type	Sample size	Sex (F : M)	Type of insulin	Mean age (years)	Outcome	Duration of study	STROBE scoring
Salman 1992	Saudi Arabia	Pre‐ and post‐design	21	13:8	Basal–bolus	11.50 ± 2.50	HbA_1c_ change, no. fasting days, hypoglycemia, hyperglycemia/ketosis	4 weeks	11
Kadiri 2001	6 Muslim countries	Case–control	64	21:43	Basal–bolus	31.8 ± 1.3	Blood glucose control, hypoglycemia	8 weeks	23
Salti 2004	13 Muslim countries	Retrospective	1,070	535:535	Non‐CSII	31.0 ± 12.70	Lifestyle changes, treatment changes, no. patients with insulin dose change, hypoglycemia, hyperglycemia/DKA	4 weeks	25
Kassem 2005	Lebanon	Pre‐ and post‐design	17	9:8	Basal–bolus	18.80 ± 4.90	HbA_1c_ change, insulin dose changes, hypoglycemia	4 weeks	19
Abbas 2008	Saudi Arabia	Case–control	9	NA	Basal–bolus vs CSII	NA	HbA_1c_ change, hypoglycemia, no. patients breaking fast	4 weeks	15
Hawli 2008	Saudi Arabia	Pre‐ and post‐design	5	1:4	CSII	16.80 ± 1.48	HbA_1c_ change, insulin dose changes, hypoglycemia, hyperglycemia/DKA	4 weeks	18
Al‐Alwan 2010	Saudi Arabia	Case–control	12	7:5	Basal–bolus	12.10 ± 1.10	HbA_1c_ change, weight change, hypoglycemia, hyperglycemia/ketosis, cholesterol level change	4 weeks	25
Al‐Khawari 2010	London, Kuwait	Pre‐ and post‐design	22	10:12	Basal–bolus vs pre‐mixed	13.90	Insulin dose changes, hypoglycemia, hyperglycemia/ketosis, weight change, no. patients breaking fast	4 weeks	26
Benbarka 2010	UAE	Pre‐ and post‐design	49	25:24	CSII	22.70 ± 7.0	Fructosamine change, no. patients breaking fast, hypoglycemia, hyperglycemia	4 weeks	25
Khalil 2012	UAE	Pre‐ and post‐design	21	11:10	CSII	29.30 ± 12.50	HbA_1c_ change, insulin dose changes, weight change, no. fasting days, hypoglycemia	8 weeks including 4 weeks before Ramadan	27
Ahmedani 2014	Pakistan	Pre‐ and post‐design	27	15:12	Non‐CSII	24.50 ± 9.8	Weight change, hypoglycemia, hyperglycemia/DKA, blood pressure change	10 weeks Including 2 weeks before and 4 weeks after Ramadan	28
Zabeen 2014	Bangladesh	Pre‐ and post‐design	33	17:16	Pre‐mixed	13.46 ± 2.06	HbA_1c_ change, insulin dose changes, weight change, hypoglycemia, hyperglycemia/ketosis/DKA (>16.7 mmol/L)	4 weeks	24
Kaplan 2015	UAE	Case–control	21	15:6	Basal–bolus vs CSII	15.0 ± 4.0	Hypoglycemia, hyperglycemia/DKA	4 weeks	19
Deeb 2016	UAE	Case–control	68	42:26	Basal–bolus vs CSII	NA	HbA_1c_ change, no. patients breaking fast, hypoglycemia, no. patients reducing insulin dose	4 weeks	23
El‐Hawary 2016	Egypt	Pre‐ and post‐design	53	29:24	Basal–bolus vs pre‐mixed	12.84 ± 1.86 13.20 ± 1.21 13.77 ± 1.79	HbA_1c_ change, fructosamine change, no. patients breaking fast, hypoglycemia, hyperglycemia/DKA, weight change, cholesterol level change	4 weeks	30
Al‐Agha 2017	Saudi Arabia	Case–control	65	29:22	Basal–bolus vs CSII	14.20 ± 2.60	HbA_1c_ change, no. fasting days, hypoglycemia, hyperglycemia	4 weeks	24
Alamoudi 2017	Saudi Arabia	Case–control	156	97:59	Basal–bolus vs CSII	23.40 ± 6.10	HbA_1c_ change, fructosamine change, no. days breaking fast, weight change, hypoglycemia, hyperglycemia/DKA	6 weeks Including 2 weeks after Ramadan	26

CSII, continuous subcutaneous insulin injection; DKA, diabetic ketoacidosis; F, female; HbA_1c_, glycated hemoglobin; M, male; NA, not available; STROBE, Strengthening the Reporting of Observational Studies in Epidemiology; UAE, United Arab Emirates.

Most studies defined hyperglycemia as a glucose level of >16.7 mmol/L. Two studies used cut‐offs of >8.3 mmol/L for fasting and >13.3 mmol/L for random blood glucose^17^, ^23^. Another two studies defined hyperglycemia using a random blood glucose level of either >15 mmol/L^11^ or >13.9 mmol/L^24^. There were four studies that did not specify any cut‐off value^2^, ^10^, ^12^, ^15^. In addition, 12 studies documented the proportion of patients who developed hyperglycemia with or without ketosis during Ramadan fasting^2^, ^10^, ^11^, ^12^, ^13^, ^14^, ^15^, ^17^, ^18^, ^21^, ^23^, ^24^.

Other outcomes reported were the number of days fasted or the proportion of patients who broke the fast^10^, ^11^, ^14^, ^17^, ^19^, ^20^, ^21^, ^22^, ^23^, ^24^, the changes in lifestyle or treatment^2^, ^8^, ^11^, ^13^, ^16^, ^18^, ^19^, ^22^, and the changes in HbA_1c_ 8, 10, 13, 14, 15, 18, 19, 20, 22, 23, 24, fructosamine^14^, ^17^, ^24^, home blood glucose levels^16^ and weight before and after Ramadan fasting^11^, ^12^, ^13^, ^14^, ^15^, ^19^, ^24^. In most studies, the duration of fasting was 4 weeks, except for three studies that extended the period to 2–4 weeks before and after the Ramadan fasting^12^, ^19^, ^24^.

A total of 1,496 patients (47.5% females) and 203 patients (39.4% females) were treated with the non‐CSII and CSII regimens, respectively. In the whole cohort, the mean ± standard deviation age and duration of diabetes was 18.6 ± 4.5 years and 86.3 ± 43.1 months, respectively. Compared with the non‐CSII‐treated group, the CSII‐treated group was older (22.9 ± 6.9 vs 17.8 ± 4.0* *years), with longer duration of diabetes (116.7 ± 66.5 vs 74.8 ± 59.2* *months) and better HbA_1c_ level (7.8 ± 1.1 vs 9.1 ± 2.0%) at baseline.

### Meta‐analysis

#### Rates of hypoglycemia, hyperglycemia and ketosis

**Figure 2:**
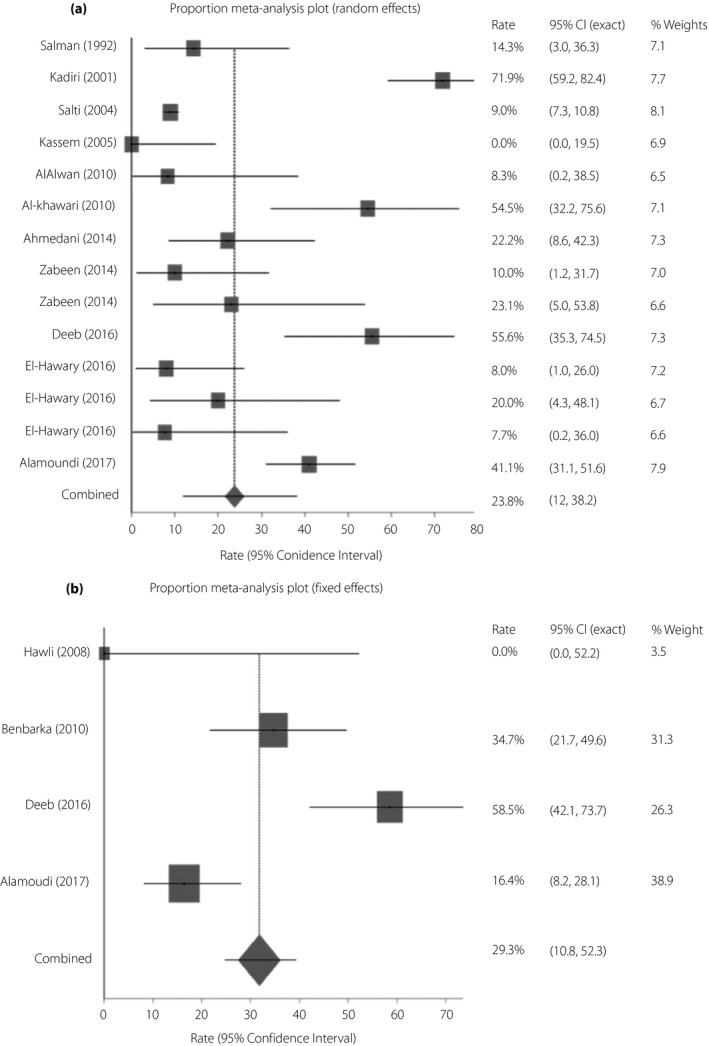
(a) Rate of hypoglycemia during Ramadan fasting among patients with type 1 diabetes on the non‐continuous subcutaneous insulin infusion regimen by random effects meta‐analysis. Rate of hypoglycemia (DerSimonian–Laird) = 23.8% (95% confidence interval [CI] 12.0–38.2), *I* ^2^ = 93.9% (95% CI 91.9–95.2). (b) Rate of hypoglycemia during Ramadan fasting among patients with type 1 diabetes on continuous subcutaneous insulin infusion by fixed effects meta‐analysis. Rate of hypoglycemia (inverse variance) = 29.3% (95% CI 10.8–52.3), *I* ^2^ = 87.1% (95% CI 62.2–93.2).

The non‐CSII‐treated group had a lower rate of hypoglycemia than the CSII‐treated group (23.8%, 95% CI 12.0–38.2 vs 29.3%, 95% CI 10.8–52.3; Figure 2a,b). During Ramadan fasting, 7.1% (95% CI 5.8–8.5) of the non‐CSII‐treated group developed severe hypoglycemia, but none from the CSII‐treated group did.

**Figure 3:**
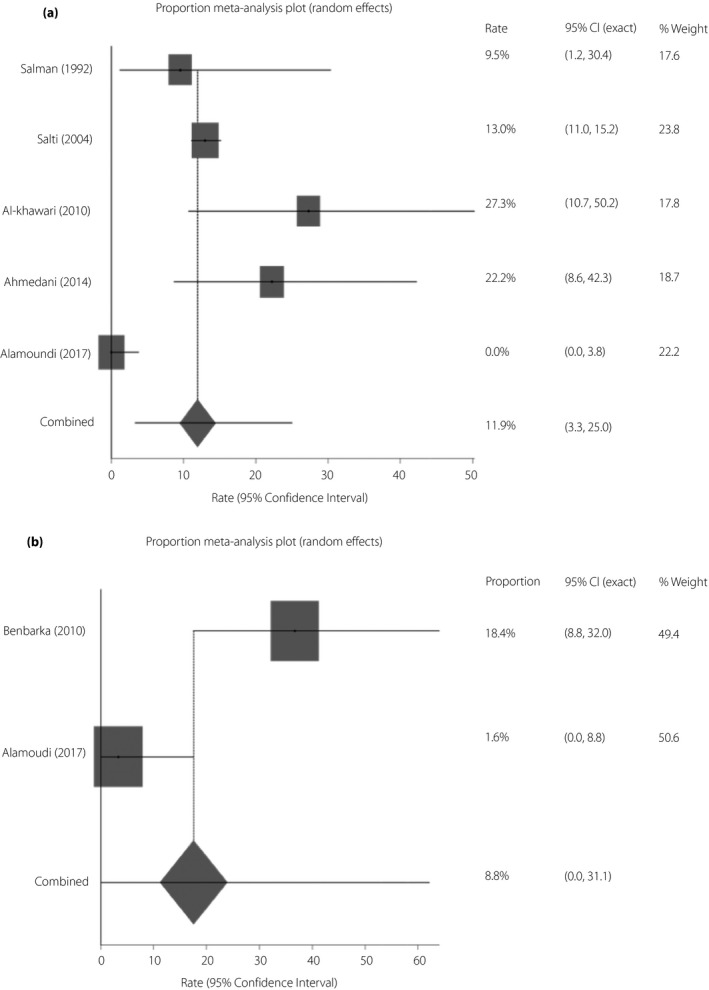
(a) Rate of hyperglycemia during Ramadan fasting among patients with type 1 diabetes on the non‐continuous subcutaneous insulin infusion regimen by random effects meta‐analysis. Rate of hyperglycemia (inverse variance) = 11.9% (95% confidence interval [CI] 3.3, 25.0), *I* ^2^ = 90.5% (95% CI 80.3–94.3). (b) Rate of hyperglycemia during Ramadan fasting among patients with type 1 diabetes on continuous subcutaneous insulin infusion by random‐effects meta‐analysis. Rate of hyperglycemia (DerSimonian–Laird) = 8.8% (95% CI 0–31.1), *I* ^2^ = 89.7% (95% CI 0–92.1).

**Figure 4:**
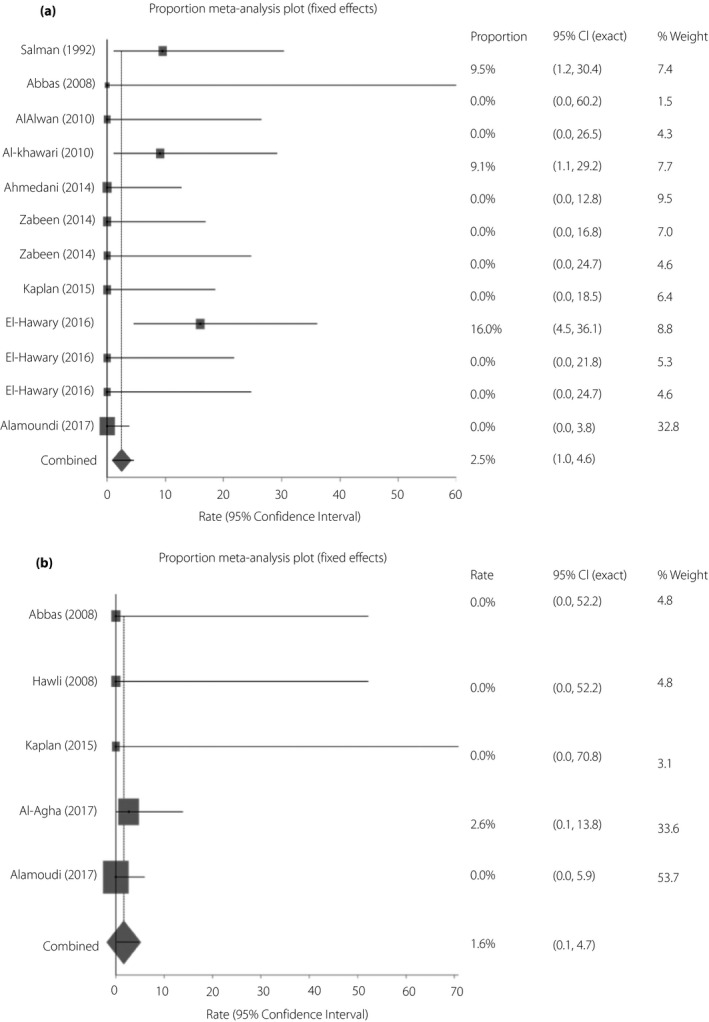
(a) Rate of ketosis during Ramadan fasting among patients with type 1 diabetes on the non‐continuous subcutaneous insulin infusion regimen by fixed effects meta‐analysis. Rate of ketosis (inverse variance) = 2.5% (95% confidence interval [CI] 1.0–4.6), *I* ^2^ = 40.7% (95% CI 0.0–68.5). (b) Rate of ketosis during Ramadan fasting among patients with type 1 diabetes on continuous subcutaneous insulin infusion by fixed effects meta‐analysis. Rate of ketosis (inverse variance) = 1.6% (95% CI 0.1–4.7), *I* ^2^ = 0% (95% CI 0.0–64.1).

Out of 12 studies, the non‐CSII‐treated group had a higher rate of hyperglycemia during Ramadan fasting compared with the CSII‐treated group (11.9%, 95% CI 3.3–25.0 vs 8.8%, 95% CI 0.0–31.1; Figure 3a,b). The corresponding figures for ketosis were 2.5% (95% CI 1.0–4.6) and 1.6% (95% CI 0.1–4.7; Figure 4a,b).

#### Breaking fast during Ramadan

**Figure 5:**
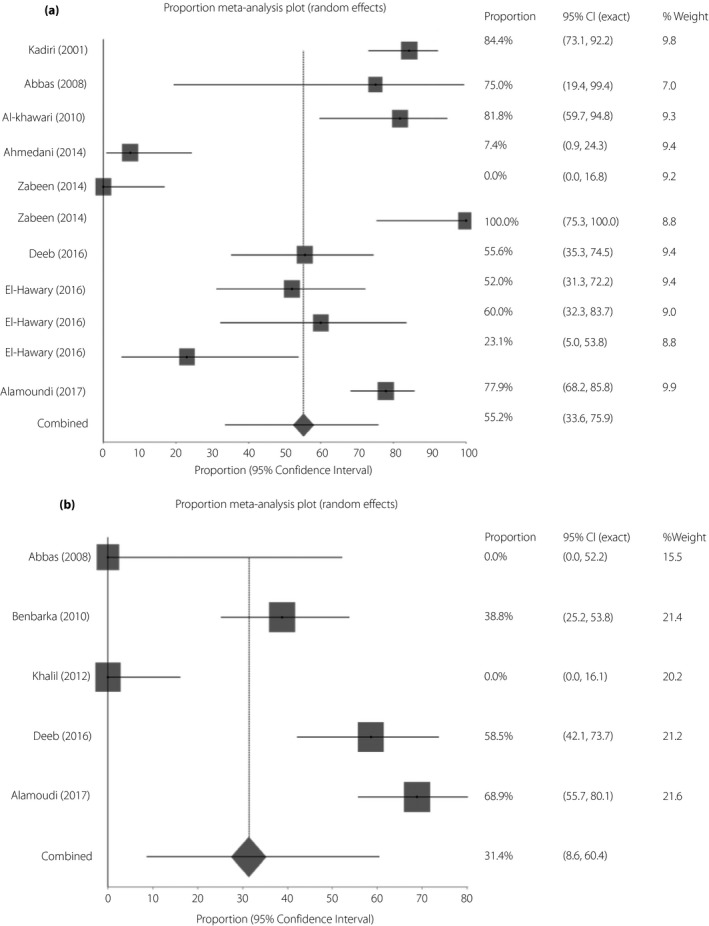
(a) Pooled proportion of breaking the fast during Ramadan fasting among patients with type 1 diabetes on non‐continuous subcutaneous insulin infusion regimen by random effects meta‐analysis. Pooled proportion (DerSimonian–Laird) = 55.2% (95% confidence interval [CI] 33.6–75.9), *I* ^2^ = 93.3% (95% CI 90.4–94.9). (b) Pooled proportion of breaking fast during Ramadan fasting among patients with type 2 diabetes on continuous subcutaneous insulin infusion by random effects meta‐analysis. Pooled proportion (DerSimonian–Laird) = 31.4% (95% CI 8.6–60.4), *I* ^2^ = 93.1% (95% CI 87.3–95.5).

More than half of the non‐CSII‐treated group (55.2%, 95% 33.6–75.9) broke their fast during Ramadan, compared with only one‐third of those treated with CSII (31.4%, 95% CI 8.6–60.4; Figure 5a,b).

#### Other outcomes

The mean HbA_1c_ after Ramadan fasting for the non‐CSII‐treated and CSII‐treated groups were 9.1 ± 2.0% and 8.0 ± 3.1%, respectively. Both groups did not have significant differences in HbA_1c_ before and after Ramadan fasting (CSII: 0.11%, 95% CI −0.08, 0.30; non‐CSII: 0.31%, 95% CI −0.01, 0.62). In three studies that reviewed changes in fructosamine pre‐ and post‐Ramadan^14^, ^17^, ^24^, the non‐CSII regimen was associated with a significant reduction in fructosamine by 1.4 μmol/L (95% CI −2.62, −0.22), but not with the CSII regimen (0.3 μmol/L, 95% CI −1.02, 0.48). There was no significant change in bodyweight (0.07 kg, 95% CI −0.12, 0.26) in the non‐CSII‐treated group after Ramadan fasting. Meta‐analysis for weight change among patients treated with CSII was not carried out, as there was only one study^24^.

A total of seven studies reported changes in total daily insulin dose during Ramadan fasting^8^, ^13^, ^14^, ^15^, ^17^, ^18^, ^19^. Both of the non‐CSII‐treated (3.4%, 95% CI −13.5, 6.8) and CSII‐treated groups (10.8%, 95% CI −25.8, 4.2) did not have a significant reduction in total daily insulin dose. The meta‐analyses of other outcomes including the number of fasting days, and the changes in lifestyle, cholesterol and blood pressure levels could not be carried out, and these are summarized in Table S1.

## Discussion

The present meta‐analysis reported that young patients with suboptimally‐controlled type 1 diabetes mellitus and treated with CSII regimen undertook Ramadan fasting with lower rates of hyperglycemia, ketosis and breaking fast compared with those treated with either premixed insulin or MDI regimen. Importantly, the CSII‐treated patients were less likely to have severe hypoglycemia requiring third‐party assistance, which is one of the major challenges in the management of diabetes during Ramadan period^25^. In contrast, we reported a higher proportion of CSII‐treated patients that experienced non‐severe hypoglycemia during Ramadan fasting than those treated with either premixed insulin or MDI regimen.

The present findings have a few clinical implications. All patients in the included studies received pre‐Ramadan counseling according to the local or international recommendations^1^. Reducing basal insulin dosage by 15–30% with appropriate adjustments of prandial insulin for MDI regimen (or reducing the Suhoor dose by 25–50% for twice‐daily premixed insulin) could have minimized the risk of mild‐to‐moderate hypoglycemia, but not severe hypoglycemia and hyperglycemia/ketosis, as shown in the present meta‐analysis^1^. Patients treated with these regimens and their families should be made aware of these potential risks if they insist on fasting during Ramadan. Given the completeness of reporting of included studies and the limited access to patient‐level data, we cannot ascertain the possible reasons explaining the higher frequency of mild‐to‐moderate hypoglycemia among CSII‐treated patients. One possibility was the CSII‐treated group had a lower pre‐Ramadan HbA_1c_ than the non‐CSII‐treated group (7.8% vs 9.1%), and thus, the CSII‐treated group might be more prone to hypoglycemia during prolonged fasting. Other physician and patient factors that were not captured, including the timing of hypoglycemia (during fasting or non‐fasting hours), the frequency of self‐monitoring of blood glucose (SMBG) or continuous glucose monitoring (CGM) with and without physicians’ feedback, the intensity of basal insulin rate adjustment, and the changes in dietary and physical activity profiles. Although the International Diabetes Federation recommends a 20–40% reduction in the basal insulin rate in the last 3–4 h of fasting for CSII‐treated patients^1^, compared with either a premixed insulin or MDI regimen, the fine‐tuning of the basal insulin rate is even more dynamic and highly individualized, of which its reduction can range from 5 to 50% during Ramadan fasting^17^, ^22^. Taken together, the present findings underscore the importance of careful patient selection for Ramadan fasting and shared decision‐making for patient‐tailored insulin therapy^1^, ^26^. CSII‐treated patients can potentially safely fast during Ramadan, provided they are well informed about the need for regular adjustments of the basal insulin rate with intensive glucose monitoring under close medical supervision.

Healthcare providers often face significant challenges in managing patients with type 1 diabetes mellitus who wish to fast during Ramadan. Exogenous insulin treatment during fasting with changes in meal timing predisposes to hypoglycemia. In addition, alterations in meal patterns, fluid intake, circadian rhythms and the sleep–wake cycle can disrupt the normal physiology of counter‐regulatory hormones (such as glucagon, cortisol and catecholamines), and therefore, increase the likelihood of hyperglycemia and diabetic ketoacidosis. To date, the International Diabetes Federation advises patients with type 1 diabetes mellitus not to undertake prolonged fasting, especially in the presence of poor glycemic control^1^. However, fasting remains a personal decision and should be respected. In real‐world settings, many patients do not perceive themselves as sick, and choose to undertake Ramadan fasting against medical advice and religious guidance^1^. In a survey involving 860 patients (9% type 1 diabetes mellitus and 91% type 2 diabetes mellitus) in Pakistan, nearly 40% of insulin‐treated patients did not carry out SMBG because of their beliefs that the finger prick could invalidate the fast^27^. Hence, in patients who insist on fasting, the CSII regimen combined with CGM can be more appealing, as this treatment plan involves less or no SMBG (depends on the types of CGM) compared with premixed insulin or MDI regimen.

Patients with type 1 diabetes mellitus have a high risk of acute complications with increasing duration of fasting. In the Epidemiology in Diabetes and Ramadan study, the respective incidence of severe hypoglycemia and hyperglycemia increased by 4.7‐ and fivefold in patients with type 1 diabetes mellitus during Ramadan fasting, compared with the non‐Ramadan period^2^. Apart from a prolonged duration of fasting after a predawn meal, patients with type 1 diabetes mellitus are more likely to develop autonomic neuropathy and have a defective counter‐regulatory response, which might explain their increased susceptibility to hypoglycemia and decreased awareness of hypoglycemia than those with type 2 diabetes mellitus 28. Of note, the types of insulin regimens can also affect the occurrence of adverse events in patients with type 1 diabetes mellitus. In a systematic review of 23 randomized controlled trials involving 976 patients with type 1 diabetes mellitus during the non‐Ramadan period, the use of the CSII regimen was associated with a 0.3% HbA_1c_ decline and decreased the risk of severe hypoglycemia compared with the MDI regimen^29^. The present findings extended to this high‐risk group, showing a reduced risk of severe hypoglycemia, hyperglycemia, ketosis and the need for breaking the fast during Ramadan.

To our knowledge, this was the first comprehensive meta‐analysis that examined the effects of Ramadan fasting among patients with type 1 diabetes mellitus treated with either the CSII or non‐CSII regimen. In addition, the present meta‐analysis included patients with suboptimal glycemic control (mean HbA_1c_ at baseline >7%) who were classified under the “very high‐risk” category and should be advised against Ramadan fasting based on the International Diabetes Federation guideline^1^. Hence, the present findings provide insights into how to advise and manage these very high‐risk patients in order to undertake Ramadan fasting as safely as possible.

The present study had several limitations. First, given the respective mean age and disease duration of 18.6 and 7 years in the whole cohort, our findings might have limited generalizability to patients who are older or with longer glycemic exposure. Second, given the lack of detailed descriptions from the included studies, we could not carry out subgroup analyses on the types of insulin regimens (premixed or MDI regimen; human insulin or insulin analogs). Third, although the need for breaking the fast was lower in the CSII‐treated group than those treated with the non‐CSII regimen, this result should be interpreted with caution. Although pre‐Ramadan counseling and written instructions on when to discontinue Ramadan fasting are offered, we acknowledge that this decision‐making process can be subjective. In an observational cohort involving 682 patients (27 patients with type 1 diabetes mellitus) in Pakistan, among six patients with type 1 diabetes mellitus who had symptomatic hypoglycemia, four of them (67%) continued to fast^12^. Fourth, the definitions of hypoglycemia used across these studies were variable, which included single glucose value ranging from <3.9 to 4.4 mmol/L with or without symptoms of hypoglycemia. In addition, these episodes of hypoglycemia were self‐reported and assessed by different SMBG and/or CGM devices with variable levels of accuracy^30^, and not confirmed by plasma glucose measurements. However, we were unable to carry out subgroup analyses using specific glucose thresholds, such as <3 mmol/L, 3 to <3.9 mmol/L and 4–4.4 mmol/L, due to a limited number of studies for each threshold. Last, given the limited access to patient‐level data, we were unable to evaluate the effects of some confounders, such as the prevalence of microvascular and macrovascular complications, the frequency and intensity of pre‐Ramadan counseling, and the adherence to diabetes in Ramadan guidelines by healthcare providers.

In conclusion, among young patients with suboptimally‐controlled type 1 diabetes mellitus who undertook Ramadan fasting, the use of the CSII regimen was associated with reduced frequencies of severe hypoglycemia, hyperglycemia and ketosis, but with a higher rate of non‐severe hypoglycemia than the non‐CSII regimen. More well‐designed studies that aim to: (i) identify patients with high glycemic variability and their appropriate treatment plans; (ii) compare the methods of intensive glucose monitoring (SMBG vs real‐time CGM vs intermittent CGM); and (iii) assess the efficacy and safety of the low‐glucose suspend insulin pumps, are crucial to providing more insights into the clinical utility of the CSII regimen in type 1 diabetes mellitus patients during Ramadan fasting.

## Disclosure

HHL has received honoraria for giving lectures from AstraZeneca, Merck Sharp & Dohme and Novartis. LLL has received research grants and/or honoraria for giving lectures from AstraZeneca, Boehringer Ingelheim, Merck Serono, Merck Sharp & Dohme, Novartis, Novo Nordisk, Sanofi Aventis and Servier. HHS and AY declare no conflict of interest.

## Supporting information


**Table S1**¦ Additional outcomes reported, but unable to carry out a pooled analysis.
