# Supplementary material for: Safety of Ramadan fasting in young patients with type 1 diabetes: A systematic review and meta‐analysis
Source: J Diabetes Investig. 2019 Apr 26;10(6):1490–501. doi: 10.1111/jdi.13054 (PMC6825934; doi:10.1111/jdi.13054)
Supplement: Supplementary file 1 — Table S1¦ Additional outcomes reported, but unable to carry out a pooled analysis. [file JDI-10-1490-s001.docx]

**Supplementary Table 1** Additional outcomes reported but unable to conduct a pool analysis

| ***Study*** | ***Type of insulin*** | ***Additional Outcomes*** | ***Finding*** |
| --- | --- | --- | --- |
| Salman 1992 | Non-CSII | Mean number of fasting days | 22.4 days |
| Kadiri 2001 | Non-CSII | Nil |  |
| Salti 2004 | Non-CSII | Lifestyle changes   - Physical activity - Sleeping duration - Food intake - Fluid intake - Sugar intake   Weight changes  Insulin dose | Unchanged 54.1%, more 10.8%, less 35.1%  Unchanged 45.0%, more 24.5%, less 30.4%  Unchanged 56.6%, more 20.3%, less 23.1%  Unchanged 56.5%, more 22.5%, less 21.1%  Unchanged 55.8%, more 23.4%, less 20.8%  Unchanged 62.5%, more 17.9%, less 19.6%  Unchanged 64.5%, more 10.7%, less 24.0%, stopped 0.8% |
| Kassem 2005 | Non-CSII | Insulin dose changes (IU/day)   - Total - Basal (Ultralante) - Prandial | Reduction of 13.4%  Reduction of 13.8%  Reduction of 12.6% |
| Abbas 2008 | Non-CSII *vs.* CSII | Insulin dose changes (%) | CSII 10 to 15% reduction of basal insulin infusion during fasting hours  Conventional insulin 10 to 20% reduction of pre-dawn insulin dose |
| Hawli 2008 | CSII | Insulin dose changes (%) | 5.5 to 25.0% reduction of basal insulin infusion in 4 out of 5 patients |
| Al-Alwan 2010 | Non-CSII | Cholesterol changes (mmol/L)   - Total - Triglyceride - LDL-cholesterol - HDL-cholesterol | 0.13 ± 0.57  0.03 ± 0.14  0.16 ± 0.48  -0.07 ± 0.19 |
| Al-Khawari 2010 | Non-CSII | Insulin dose changes | 8 to 16% reduction in total insulin dose in 3 patients |
| Benbarka 2010 | CSII | Nil |  |
| Khalil 2012 | CSII | Insulin dose changes   - Total (IU/day) - Basal (IU/day) - Prandial/total (%) - Percentage reduction   BMI change (kg/m^2^)  Median number of fasting days | -0.84 ± 9.71  -1.4 ± 4.0  3.2 ± 7.8  5 to 20% reduction of basal insulin during daytime  -0.1 ± 1.0  29 days |
| Ahmedani 2014 | Non-CSII | Blood pressure change (mmHg)   - Systolic - Diastolic | 107.36 ± 14.84 before, 108.42 ± 13.85 after  75.50 ± 15.03 before, 74.75 ± 7.52 after |
| Zabeen 2014 | Non-CSII | Nil |  |
| Kaplan 2015 | Non-CSII *vs.* CSII | Nil |  |

| ***Study*** | ***Type of insulin*** | ***Additional Outcomes*** | ***Finding*** |
| --- | --- | --- | --- |
|  |  |  |  |
| Deeb 2016 | Non-CSII *vs.* CSII | Number of patients reducing basal insulin dose | 24 patients by 25%, 8 patients by 20%, 3 patients by 10% |
| El-Hawary 2016 | Non-CSII | Cholesterol change (mmol/L)   - Total - Triglyceride - HDL-cholesterol - LDL-cholesterol | 5.34% to 31.0% increment  3.62% reduction to 27.17% increment  5.04% reduction to 34.70% increment  5.89% to 35.06% increment |
| Al-Agha 2017 | Non-CSII *vs*. CSII | Number of fasting days | 67% of total fasting days (752 days) out of 1153 days |
| Alamoudi 2017 | Non-CSII *vs.* CSII | Nil |  |

**Legend** BMI, body mass index; CSII, continuous subcutaneous insulin infusion; HDL-cholesterol, high-density lipoprotein cholesterol; LDL-cholesterol, low-density lipoprotein cholesterol
